# Mixed-Stacking Few-Layer Graphene as an Elemental Weak Ferroelectric Material

**DOI:** 10.1021/acs.nanolett.2c04723

**Published:** 2023-05-09

**Authors:** Aitor Garcia-Ruiz, Vladimir Enaldiev, Andrew McEllistrim, Vladimir I. Fal’ko

**Affiliations:** † School of Physics and Astronomy, University of Manchester, Oxford Road, Manchester M13 9PL, U.K.; ‡ National Graphene Institute, University of Manchester, Oxford Road, Manchester M13 9PL, U.K.; ¶ Henry Royce Institute for Advanced Materials, University of Manchester, Oxford Road, Manchester M13 9PL, U.K.

**Keywords:** Ferroelectricity, graphene, rhombohedral graphite, twin boundary, twistronics, screening

## Abstract

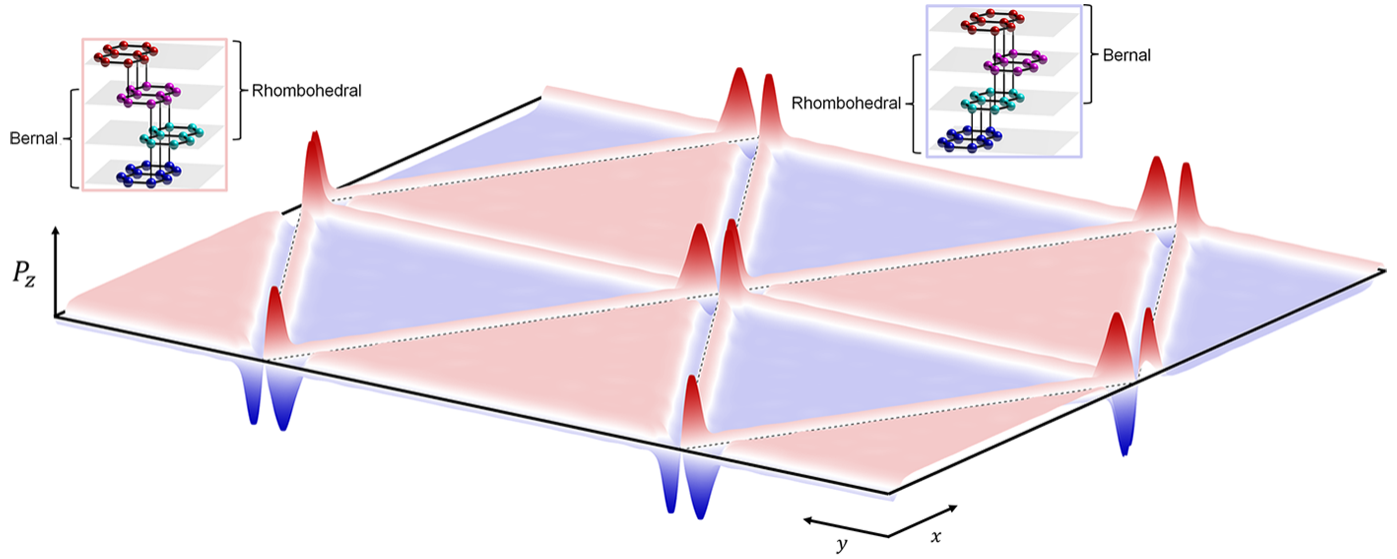

Ferroelectricity (ValasekJ.Phys. Rev.1921, 17, 475), a spontaneous formation of electric polarization, is a solid state phenomenon, usually, associated with ionic compounds or complex materials. Here we show that, atypically for elemental solids, few-layer graphenes can host an equilibrium out-of-plane electric polarization, switchable by sliding the constituent graphene sheets. The systems hosting such effect include mixed-stacking tetralayers and thicker (5–9 layers) rhombohedral graphitic films with a twin boundary in the middle of a flake. The predicted electric polarization would also appear in marginally (small-angle) twisted few-layer flakes, where lattice reconstruction would give rise to networks of mesoscale domains with alternating value and sign of out-of-plane polarization.

Ferroelectricity, a spontaneous electric polarization in the absence of an external electric field, is a phenonmenon observed and thoroughly investigated in a broad range of solids.^2−4^ Caused by charge transfer between constituent atoms in a unit cell, up to now, ferroelectric polarization was observed only in chemically complex compounds, such as Rochelle salts,^1^ Pb[Zr_*x*_Ti_1–*x*_]O_3_,^5^ BaTiO_3_,^6^ etc.^7−11^ Here, we show that there is one exception from this common rule, namely, several structural allotropes of multilayer graphene.

The above statement is based on a theoretical study of various multilayer graphene structures with different stacking orders which, as an elemental material, is nonpolar: its intralayer bonding is dominantly covalent, whereas the interlayer adhesion has a van der Waals nature. There are two commonly studied multilayer graphene systems: thin films of Bernal graphite^12,13^ and rhombohedral (ABC) graphite.^14^ Those two possess inversion symmetry, or *z* → −*z*, respectively, which prohibit spontaneous ferroelectric polarization. However, graphitic films with mixed Bernal and rhombohedral stackings lack such symmetries, removing constraints on the formation of an out-of-plane electric dipole. One example of such a structure is a thin film of rhombohedral graphite with a twin boundary inside it,^15^ which consists of two ABC parts with *n* and *m* layers (*n* ≠ *m*), rotated by 180° with respect to each other, held together by an ABA-trilayer (see Figure 1).

**Figure 1:**
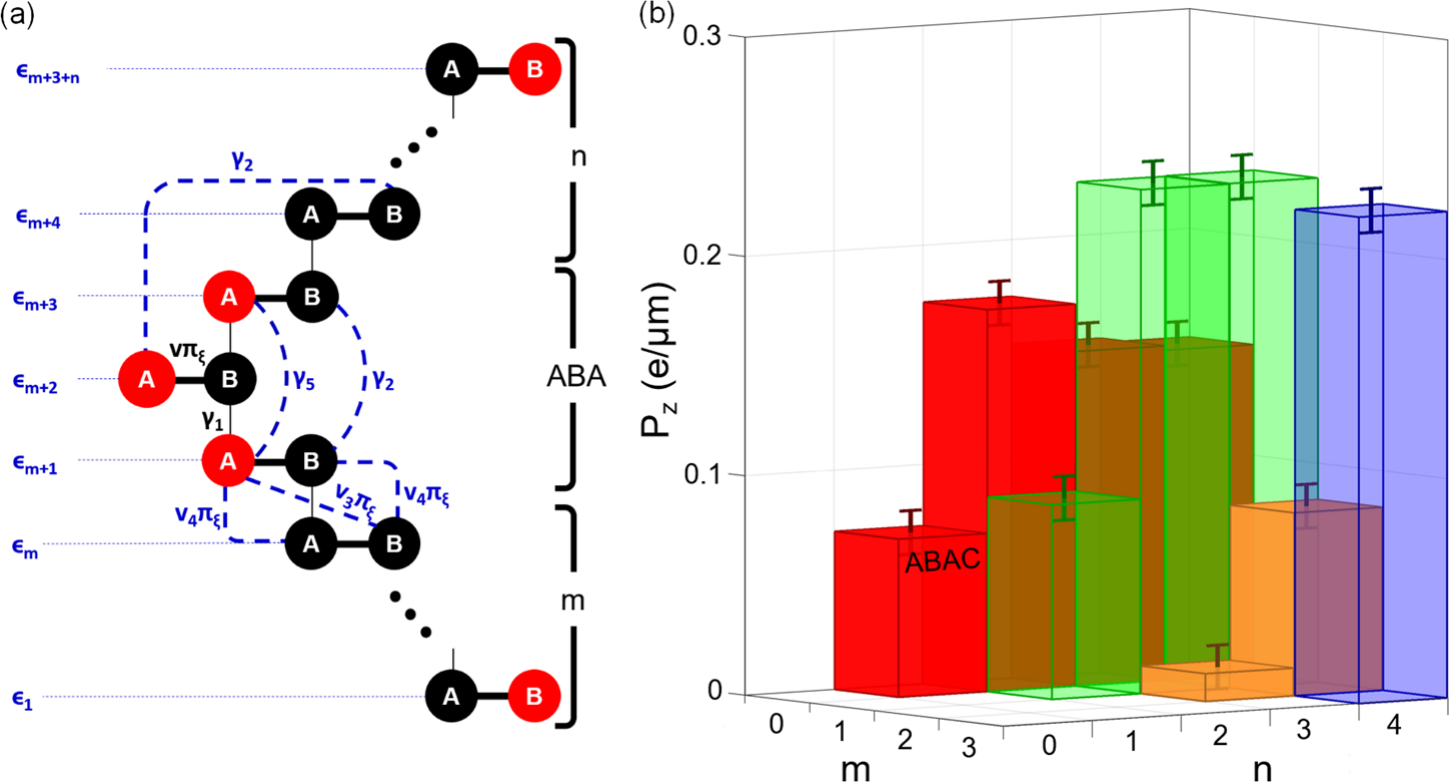
Sketch of a twinned rhombohedral graphitic film, highlighting in dashed lines the SWMcC couplings, with a radius proportional to the number of dimer bonds and showing in red the low-energy orbitals (three non-dimer bonds and the antisymmetric combination of A-sublattice orbitals of the layers adjacent to the twin boundary). The right panel shows the dependence of the ferroelectric polarization density as a function of thicknesses *n* and *m* of twinned layers.

In the following section, we study *n*ABA*m*, with *n* ≠ *m*, and demonstrate that they exhibit a weak spontaneous out-of-plane electric polarization, *P*_*z*_, at zero doping. We determine the roles of parameters in the full multiparameter Slonczewski–Weiss–McClure (SWMcC) Hamiltonian that are related to the asymmetries that permit a finite *P*_*z*_. Importantly, we find that self-consistently implemented screening of internal electric field not only strongly reduces *P*_*z*_ magnitude but also changes its sign for some structures, which is a result of different interlayer charge redistributions, caused individually by each symmetry-breaking term in the bare SWMcC Hamiltonian. For tetralayers, the known values and signs of SWMcC parameters^16^ are such that the largest contributions they produced individually mostly cancel each other, making the result sensitive to the precise choice of parameters. At the same time, for marginally twisted tetralayers, where lattice relaxation leads to the formation of equilibrium stacking domains (where polarization is small), the domain walls host seeds of different stacking arrangements for which such cancellation does not occur leading to an order of magnitude larger local *P*_*z*_ values.

Below, we use the full Slonczewski–Weiss–McClure (SWMcC) model of graphite films discussed in ref (15). This model accounts for couplings sketched in Figure 1, which include both the closest and next-neighbor hoppings, and it is implemented in the framework of a hybrid **k**·**p**-tight-binding model,^17^ using a Hamiltonian, , specified in the Supporting Information. The diagonalization of  gives the dispersions, ε_β_(**p**), and wave functions, ψ_β,**p**_^α_*L*_^, of bands β in the multilayer, which we use to compute both on-layer electron densities, *n*_*L*_, and electric polarization:
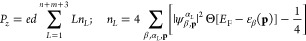
1Here *e* is the electron charge, *d* ≈ 3.35 Å the interlayer distance, and “*L*” the layer index, and α_*L*_ = *A*_*L*_, *B*_*L*_ sublattice indices. When computing the electron densities, *n*_*L*_, we take account of the screening the potential they generate on each individual layer, as
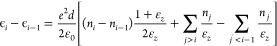
2where ε_*z*_ ≈ 2.6 is an effective out-of-plane dielectric permittivity of graphene stacks determined by the polarizability of carbon atoms.^18^

Among all SWMcC couplings, next-neighbor hoppings γ_2_, γ_5_, and *v*_4_, together with the energy difference between dimer and non-dimer sites, Δ′, are most important for a nonzero value of the ferroelectric polarization, *P*_*z*_, to form across the structure. This is because such couplings break a hidden electron–hole symmetry,^a^ ( is a unitary matrix equal to direct product of *n*+3+*m*-rank identity and third Pauli matrices, respectively), characteristic of the “reduced” models of graphene limited to the closest neighbor couplings only. The on-layer potentials in eq 2 induced by the screening can also break all symmetries, which will be important for understanding the result of self-consistent analysis.

For a detailed quantitative self-consistent analysis of spontaneous electric polarization *P*_*z*_, we implement the following steps. First, we compute the electric polarization induced by one of the four symmetry-breaking terms in the SWMcC Hamiltonian for each of the considered structures, both with and without self-consistent implementation of screening. Then, we check that the cumulative effect of all the terms in the full SWMcC model can be approximated as a sum of the individual symmetry-breaking contributions as
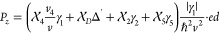
3Here,  are dimensionless factors for which values for the ABCB tetralayer are listed in Table 1.

**Table 1:** Numerical Values for the Parameters  in Eq 3 and Their Contribution towards the Polarization, with and without Including the Screening Effects of On-Layer Charge Redistribution^a^

	*P*_*z*_^*u*^ (*e*/μm)		coeffs.		*P*_*z*_ (*e*/μm)	ϵ_2_ – ϵ_1_ (meV)	ϵ_3_ – ϵ_2_ (meV)	ϵ_4_ – ϵ_3_ (meV)
total	0.50				–0.07			
*v*_4_ = 0.022*v*	0.09	0.036	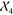	0.009	0.02	–1.332	1.323	–0.418
Δ′ = 25 meV	–0.33	–0.045	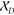	–0.032	–0.23	–5.607	5.631	0.716
γ_2_ = –17 meV	0.28	–0.058	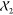	0.016	–0.08	1.403	–1.959	2.158
γ_5_ = 38 meV	0.45	0.041	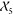	0.019	0.21	1.498	–1.567	–1.404
	unscreened			self-consistently screened				

a In the last four rows, we also include the on-layer potentials. To evaluate the dependence with respect to each parameter, we use a Hamiltonian model where all other SWMcC parameters were set to zero except *v* = 1.02 × 10^6^ m/s and γ_1_ = 390 meV.

Accounting for the charge redistribution self-consistently is an important part of the presented calculations. Taking into account redistribution of charges produced by screening of electric fields attributed to polarization not only reduces the value of *P*_*z*_ by 1 order of magnitude, but also can change the sign of some of the individual symmetry-breaking contributions, when compared with the unscreened case. We investigate numerically this effect by comparing the values for  in ABCB graphenes, with and without the self-consistent implementation of screening in Table 1. In the absence of screening (ϵ_*i*_ = 0), we obtain a value of *P*_*z*_ ≈ 0.5 *e*/μm, whereas implementing screening changes this value to *P*_*z*_ = −0.07 *e*/μm. This is because the two largest contributions to the ferroelectric polarization, coming from  and , have opposite signs and nearly cancel each other for the choice of SWMcC based on ref (16), leaving γ_2_ to define the value of *P*_*z*_, so that the overall result changes sign after the implementation of screening. We suggest that such a sensitivity of the computed values of *P*_*z*_ to the input SWMcC parameters may be used to further narrow down their choices by comparing the computed *P*_*z*_ with the experimentally measured polarization of tetralayers. We find that screening is equally important for the analysis of polarization in thicker films, though the above-mentioned cancellation does not occur for all thicknesses. In Figure 1, we show the computed values of *P*_*z*_ in various *n*ABA*m* structures with *m* > *n* (the mirror-symmetric *m*ABA*n* configurations have the same magnitude of *P*_*z*_ but opposite sign).

While the above-described twinned rhombohedral graphitic films may appear naturally among flakes exfoliated from bulk material, one can try to build weakly ferroelectric multilayers by a twistronic assembly of thinner flakes.^19−23^ A small misalignment of crystalline axes of the assembled graphene flakes, unavoidable in the mechanical transfer, leads to a long period variation of stacking, known as moiré structure, which upon lattice relaxation (promoting energetically preferred local stacking order) results in a network of domains with AB and BA interlayer preferences at the twisted interface.

Here, we consider structures with graphene monolayers small-angle twisted with respect to Bernal and rhombohedral trilayer graphene (3ABA+1 and 3ABC+1, respectively) and a twisted double-bilayer (2AB+2BA). At the twisted interface, the moiré superlattice undergoes lattice reconstruction promoting formation of triangular domain arrays with local AB and BA stackings, shown in Figure 2, which are separated by a network of domain walls (DWs). We model lattice reconstruction in the structures following the approach of refs (24 and 25) to find local deformations of lattices in top/bottom flakes, **u**_t/b_, that minimize (via domain formation) a sum of elastic and interlayer adhesion energies (see Supporting Information).

**Figure 2:**
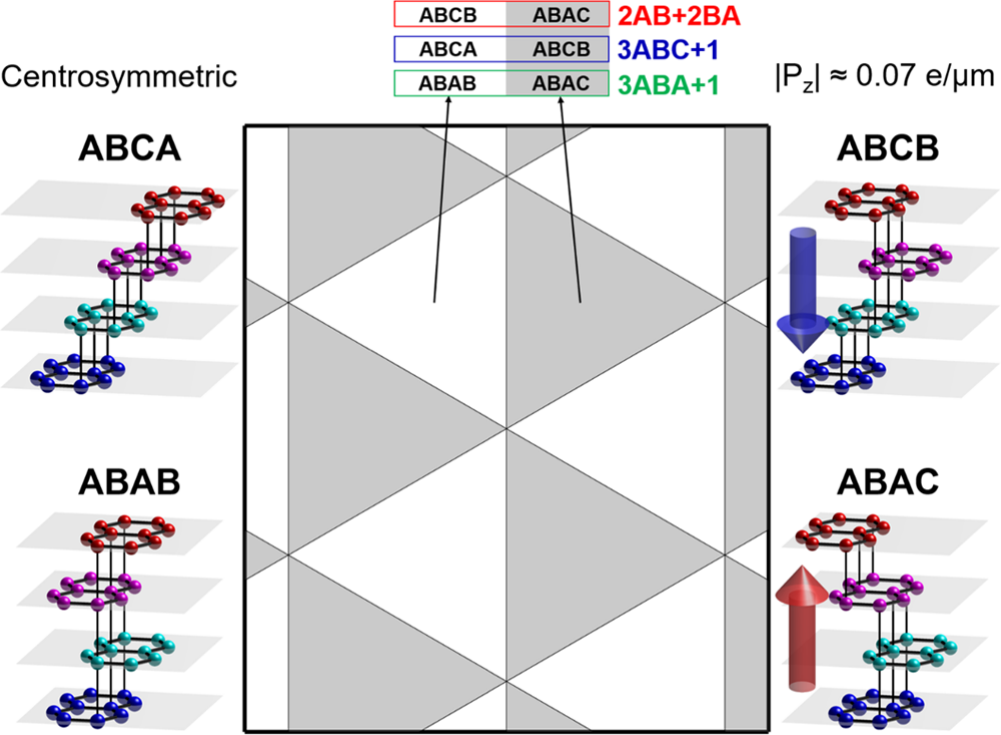
An array of triangular domains in a marginally twisted tetralayer graphene (2AB+2BA, 3ABA+1 and 3ABC+1), which we expect to form by lattice reconstruction of a long-period moiré superlattice. Domain stackings with and without ferroelectric charge transfer for various twisted tetralayer structures are shown on the right and left-hand side sketches, respectively.

Then, we find the ferroelectric polarization of small-angle twisted structures using local stacking approximation. That is, we calculate the polarization based on a Hamiltonian shown in Methods, describing the aligned tetralayer films with an in-plane offset **r**_0_ = θ*ẑ* × **r** + **u**_t_ – **u**_b_ between constituent parts. As a reference, we set **r**_0_ = 0 for ABBA-stacking in 2AB+2BA, ABAA-stacking in 3ABA+1, and ABCC-stacking in 3ABC+1 areas. Having calculated *P*_*z*_ with a self-consistent analysis of screening and on a sufficiently dense grid of interlayer offsets **r**_0_, we interpolate it in the form of a harmonic series,
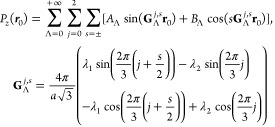
4where Λ labels reciprocal lattice vectors of graphene in the ascending order of the lengths (see Table 2). Note that mirror reflection of AB and BA bilayers in 2AB+2BA films translates to symmetry properties of *P*_*z*_(**r**_0_). In particular, for a reference frame with *x*-axis along zigzag and *y*-axis along armchair, *P*_*z*_(*x*_0_, *y*_0_) = *P*_*z*_(−*x*_0_, *y*_0_), as the zigzag axis is perpendicular to the vertical mirror symmetry plane conserving *P*_*z*_, and *P*_*z*_(*x*_0_, *y*_0_) = −*P*_*z*_(*x*_0_, −*y*_0_), as mirror reflection in the armchair axis is equivalent to exchange of AB ↔ BA stackings in each bilayer and consequently leads to inversion of *P*_*z*_. This requires *B*_Λ_ = 0 in eq 4 for 2AB+2BA films, whereas the other Fourier coefficients are listed in Table 2.

**Table 2:** Coefficients of the Fourier Expansion in Eq 4 for the Three Stacking Configurations Analyzed

		AB+BA	ABA+1		ABA+1	
Λ	{λ_1_, λ_2_}	*A*_Λ_ (*e*/μm)	*A*_Λ_ (*e*/μm)	*B*_Λ_ (*e*/μm)	*A*_Λ_ (*e*/μm)	*B*_Λ_ (*e*/μm)
0	{0, 0}			0.0384		0.0243
1	{1, 0}	0.0626	–0.0293	–0.1231	0.0488	–0.1599
2	{2, 1}	0	0	–0.0917	0	–0.1015
3	{2, 0}	0.0847	–0.0320	0.0927	0.0621	0.0946
4	{3, 1}	0.0147	–0.0045	–0.0319	0.0096	–0.0369
5	{3, 0}	0	–0.0067	0.0307	0.0015	0.0140
6	{4, 2}	0	0	–0.0335	0	–0.0208
7	{4, 1}	0.0118	–0.0039	0.0151	0.0052	0.0083
8	{4, 0}	0.0076	–0.0057	0.0024	0.0102	–0.0036
9	{5, 2}	0.0015	0.0013	–0.0130	–0.0021	–0.0118
10	{5, 1}	–0.0002	–0.0017	0.0189	0.0001	0.0013
11	{5, 0}	0.0062	–0.0025	0.0158	0.0032	0.0009
12	{6, 3}	0	0	–0.0151	0	–0.0060
13	{6, 2}	0.0013	0.0004	0.0033	–0.0005	–0.0002
14	{6, 1}	0.0035	–0.0025	0.0011	0.0041	–0.0014
15	{6, 0}	–0.0004	–0.0001	0.0042	–0.0002	–0.0024
16	{7, 3}	0.0009	0.0023	–0.0071	–0.0026	–0.0046
17	{7, 2}	0.0001	–0.0010	0.0036	–0.0001	–0.0007
18	{7, 1}	0.0024	–0.0007	0.0015	0.0013	–0.0004
19	{8, 4}	0	0	–0.0081	0	–0.0025
20	{7, 0}	0.0019	–0.0014	0.0012	0.0018	–0.0006

In Figure 3, we illustrate the resulting real space distributions of the ferroelectric polarization in a representative region of moiré superlattice for each of the considered tetralayer films. The mirror reflection in armchair axes prescribes zero polarization for DWs in 2AB+2BA films, shown in Figure 2, oriented along armchair crystallographic axes and separating domains with opposite polarization, *P*_*z*_ = ±0.07 *e*/μm. This would correspond to the transfer of ∼10^11^ cm^–2^ electron density between the tetralayer film surfaces. Note that close to domain corners polarization reverses sign and its magnitude become four times higher than those in the middle of domains.

**Figure 3:**
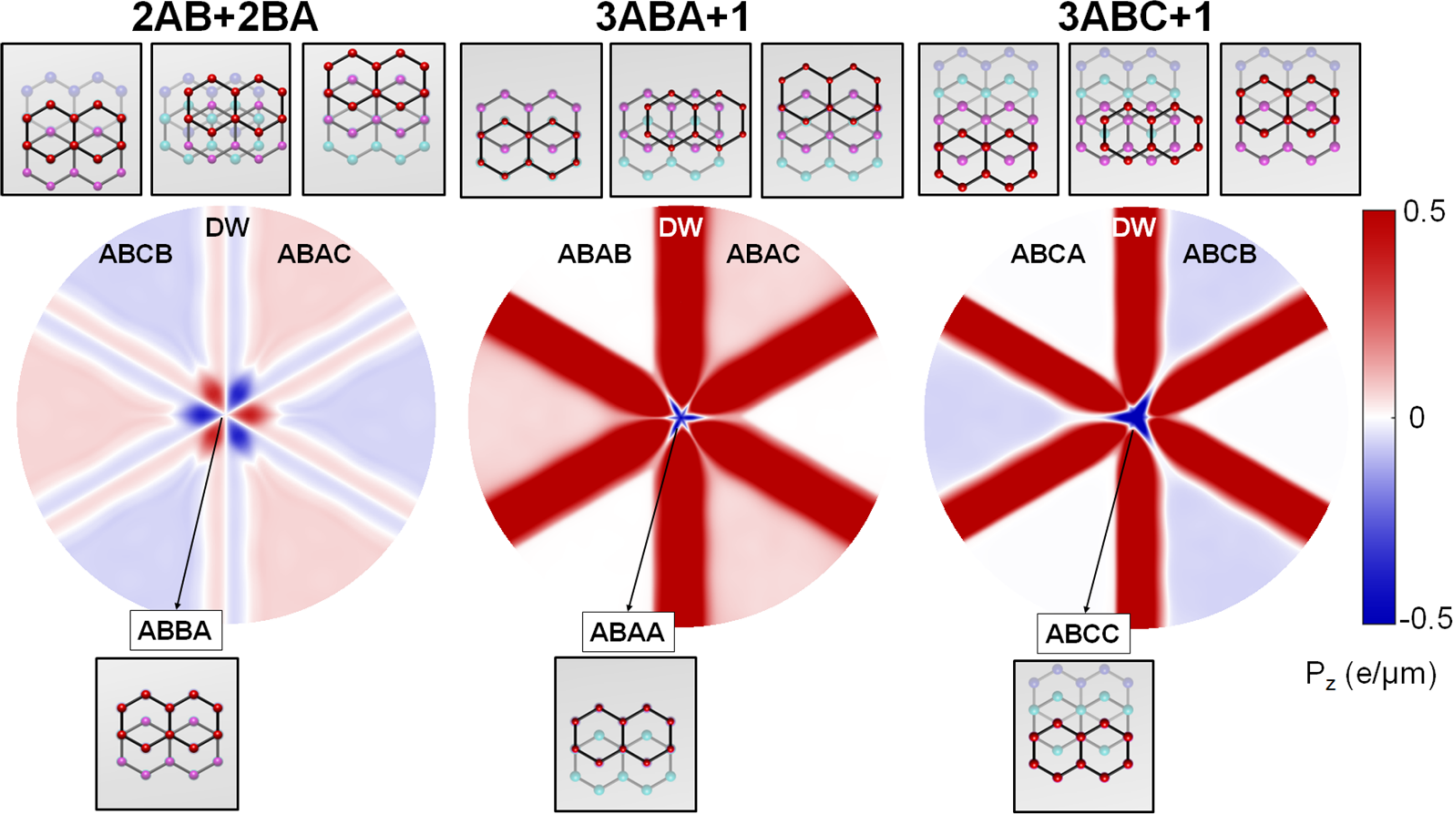
Ferroelectric polarization around a circular area of 1 μm of diameter, centered at the intercross of triangular networks of domain walls, shown in Figure 2. The insets represent the top view of the local stacking configuration at the domains, the domain walls, and the center of the intercross of triangular network of domain walls. .

DW intercrossings possess opposite polarization compared to the inner part of the DW. In contrast, supercells of 3ABA+1 (3ABC+1) twisted tetralayers contain one inversion symmetric domain, ABAB (ABCA) (see Figure 2), where ferroelectric polarization is forbidden by symmetry and another, ABAC (ABCB) domain (as in 2AB+2BA films), which carries an out-of-plane polarization. We note that in 3ABA+1 (3ABC+1) tetralayers DWs and their intercrossings host even an order of magnitude stronger local vertical charge transfer than in the inner areas of the domains.

The spontaneous out-of-plane polarization that we predict here for few-layer rhombohedral graphene structures is a feature induced by an asymmetrically placed twin boundary which breaks inversion and mirror symmetry in the system, and we trace it to the effect of individual terms in the full Sloczewski–Weiss–McClure model of graphite involved with such asymmetries and responsible for electron–hole symmetry breaking of the single-particle spectra.

The reported calculations show that the size and orientation of *P*_*z*_ are critically affected by the intrinsic screening inside the graphitic films, leading to the opposite signs of *P*_*z*_ in tetralayers analyzed with and without screening and much smaller values, about 1 order of magnitude smaller than those experimentally reported in WTe_2_,^26^ MoS_2_,^11^ or hBN.^27^ We also note that in marginally twisted multilayers, the largest local polarization appears to be at domain walls and the near the nodes of domain wall networks. Overall, such sensitivity to the choice of the values of input parameters in the tight-binding model of graphite, number of layers in the film, and variation across the moiré pattern in marginally twisted structures may be used to experimentally refine the parametrization of the Sloczewski–Weiss–McClure model.

## Data Availability

The data that support the plots in the manuscript are available from the corresponding authors upon reasonable request.

## Supporting Information Available

Methods used to produce the data of the text, including: description of the Hamiltonian of rhombohedral graphite with a twin boundary, description of the Hamiltonian of tetralayer graphite with one interface laterally shifted by an arbitrary amount **r**_0_, analysis of the area in reciprocal space required for convergence, description of the method used to account for electrostatic screening, analysis of the dependence of *P*_*z*_ and *P*_*z*_^*u*^ on each of the SWMcC parameters, and description of the method used to account for lattice relaxation (PDF)

## Footnotes

a Applying  to Hamiltonian, , we change signs of all of its matrix elements except those on the diagonal of matrices *H*_g_^*s*,*b*^, *V*, *W*, and *W̃* being proportional to either of the parameters *v*_4_, Δ′, γ_2_, γ_5_. Therefore, for *v*_4_ = Δ′ = γ_2_ = γ_5_ = 0, the Hamiltonian possesses the hidden electron–hole symmetry , which results in *P*_*z*_ = 0. This is because at zero doping polarizations given by electron and hole parts of the spectrum should be opposite, whereas the electron–hole symmetry equalizes them requiring a zero value for both.
