# Supplementary material for: Mixed-Stacking Few-Layer Graphene as an Elemental Weak Ferroelectric Material
Source: Nano Lett. 2023 May 9;23(10):4120–5. doi: 10.1021/acs.nanolett.2c04723 (PMC10214444; doi:10.1021/acs.nanolett.2c04723)
Supplement: Supplementary file 1 — nl2c04723_si_001.pdf [file nl2c04723_si_001.pdf]

Supporting information for "Mixed-stacking few-layer graphene as an elemental weak ferroelectric material"

Aitor Garcia-Ruiz,[*,†,‡] Vladimir Enaldiev,[†,‡] Andrew McEllistrim,[†,‡] and Vladimir I. Fal'ko[†,‡,¶]

†*School of Physics and Astronomy, University of Manchester, Oxford Road, Manchester, M13 9PL, UK*

‡*National Graphene Institute, University of Manchester, Oxford Road, Manchester, M13 9PL, UK*

¶*Henry Royce Institute for Advanced Materials, University of Manchester, Oxford Road, Manchester, M13 9PL, UK*

E-mail: altor.garcia-ruiz@manchester.ac.uk

Hamiltonian of rhombohedral graphite with a twin boundary

To construct the Hamiltonian of rhombohedral graphite with a twin-boundary fault, we incorporate all possible couplings described in Figure 1 of the main text. In the basis of A and B sublattice amplitudes and from the bottommost to the topmost layers, $\{\psi_{\beta,\boldsymbol{p}}^{A_1}, \psi_{\beta,\boldsymbol{p}}^{B_1}, ..., \psi_{\beta,\boldsymbol{p}}^{B_{n+m+3}}\}$, this Hamil-

tonian takes the form

$$\mathcal{H} = \begin{pmatrix} H_{\mathrm{g}}^s & V & W & \cdots & 0 & \cdots & 0 & 0 & 0 \\ V^\dagger & H_{\mathrm{g}}^b & V & \cdots & 0 & \cdots & 0 & 0 & 0 \\ W^\dagger & V^\dagger & H_{\mathrm{g}}^b & \cdots & 0 & \cdots & 0 & 0 & 0 \\ \vdots & \vdots & \vdots & \ddots & \vdots & \vdots & \vdots & \vdots & \vdots \\ 0 & 0 & 0 & \cdots & \mathcal{H}_{\mathrm{ABA}} & \cdots & 0 & 0 & 0 \\ \vdots & \vdots & \vdots & \cdots & \vdots & \ddots & \vdots & \vdots & \vdots \\ 0 & 0 & 0 & \cdots & 0 & \cdots & H_{\mathrm{g}}^b & V^\dagger & W^\dagger \\ 0 & 0 & 0 & \cdots & 0 & \cdots & V & H_{\mathrm{g}}^b & V^\dagger \\ 0 & 0 & 0 & \cdots & 0 & \cdots & W & V & H_{\mathrm{g}}^s \end{pmatrix}, \tag{1}$$

$$H_{\mathrm{g}}^s = H_g + \begin{pmatrix} \Delta' & 0 \\ 0 & 0 \end{pmatrix}, \quad H_{\mathrm{g}}^b = H_g + \Delta'\hat{1}_2, \quad H_g = v \begin{pmatrix} 0 & \pi_\xi^* \\ \pi_\xi & 0 \end{pmatrix},$$

$$V = \begin{pmatrix} -v_4\pi_\xi & \gamma_1 \\ -v_3\pi_\xi^* & -v_4\pi_\xi \end{pmatrix}, \quad W = \begin{pmatrix} 0 & 0 \\ \gamma_2/2 & 0 \end{pmatrix}, \quad \pi_\xi \equiv \xi p_x + i p_y,$$

$$\mathcal{H}_{ABA} = \begin{pmatrix} H_{\mathrm{g}}^b & V & \tilde{W} \\ V^\dagger & H_{\mathrm{g}}^b - \sigma_z\Delta' & V^\dagger \\ \tilde{W}^\dagger & V & H_{\mathrm{g}}^b \end{pmatrix}, \quad \tilde{W} = \begin{pmatrix} \gamma_5/2 & 0 \\ 0 & \gamma_2/2 \end{pmatrix},$$

where we introduce the Sloczewski-Weiss-McClure (SWMcC) parameters[1–3], $v \approx 1.02 \cdot 10^6$ m/s, $\gamma_1 = 390$ meV, $v_3 \approx 0.1v$, $v_4 \approx 0.022v$, $\Delta' = 25$ meV, $\gamma_2 = -17$ meV and $\gamma_5 = 38$ meV[4], and $\xi = \pm 1$ is the valley index.

Hamiltonian of tetralayer graphite with a marginally twisted interface

Local stacking characterised by an interlayer offset, $\boldsymbol{r}_0$ at the twsited interface. The following Hamiltonians generalise those derived in Ref.[5]. For 2AB+2BA, Hamiltonian reads

$$\mathcal{H}_{AB+BA} = \begin{pmatrix} H_{\mathrm{g}}^s & V & \mathcal{W}_{\mathrm{AB+1}}(\boldsymbol{r}_0) & 0 \\ V^\dagger & H_{\mathrm{g}}^s - \sigma_z\Delta' + \mathcal{E}_b & \mathcal{V}(\boldsymbol{r}_0) & \mathcal{W}_{\mathrm{1+BA}}(\boldsymbol{r}_0) \\ \mathcal{W}_{\mathrm{AB+1}}^\dagger(\boldsymbol{r}_0) & \mathcal{V}^\dagger(\boldsymbol{r}_0) & H_{\mathrm{g}}^s - \sigma_z\Delta' + \mathcal{E}_t & V^\dagger \\ 0 & \mathcal{W}_{\mathrm{1+BA}}^\dagger(\boldsymbol{r}_0) & V & H_{\mathrm{g}}^s \end{pmatrix} \tag{2}$$

$$\mathcal{V}(\boldsymbol{r}_0) = \sum_{j=0}^{2} \left\{ \left[\frac{\gamma_1}{3} - \frac{2v_4\hbar}{3K}\boldsymbol{k}\cdot\boldsymbol{K}_\xi^{(j)}\right] \begin{pmatrix} 1 & e^{i\xi\frac{2\pi}{3}j} \\ e^{-i\xi\frac{2\pi}{3}j} & 1 \end{pmatrix} \right.$$
$$\left. + \frac{\xi 2(v_3 - v_4)\hbar}{3K}\left[\boldsymbol{k}\times\boldsymbol{K}_\xi^{(j)}\right]_z \begin{pmatrix} 0 & ie^{i\xi\frac{2\pi}{3}j} \\ -ie^{-i\xi\frac{2\pi}{3}j} & 0 \end{pmatrix} \right\} e^{-i\boldsymbol{K}_\xi^{(j)}\cdot\boldsymbol{r}_0},$$

$$\mathcal{W}_{\mathrm{AB+1}}(\boldsymbol{r}_0) = \frac{1}{6}\sum_{j=0}^{2} \begin{pmatrix} \gamma_5 e^{-i\xi\frac{2\pi}{3}j} & \gamma_5 \\ \gamma_2 e^{+i\xi\frac{2\pi}{3}j} & \gamma_2 e^{-i\xi\frac{2\pi}{3}j} \end{pmatrix} e^{-i\boldsymbol{K}_\xi^{(j)}\cdot\boldsymbol{r}_0},$$

$$\mathcal{W}_{\mathrm{1+BA}}(\boldsymbol{r}_0) = \frac{1}{6}\sum_{j=0}^{2} \begin{pmatrix} \gamma_5 e^{+i\xi\frac{2\pi}{3}j} & \gamma_2 e^{-i\xi\frac{2\pi}{3}j} \\ \gamma_5 & \gamma_2 e^{+i\xi\frac{2\pi}{3}j} \end{pmatrix} e^{-i\boldsymbol{K}_\xi^{(j)}\cdot\boldsymbol{r}_0},$$

$$\mathcal{E}_{b/t} = \frac{2\Delta'}{3}\hat{1}_2 + \frac{\Delta'}{9}\sum_{\boldsymbol{G}} e^{i\boldsymbol{G}\cdot\boldsymbol{r}_0} \begin{pmatrix} 1 + e^{\pm i\boldsymbol{G}\cdot\boldsymbol{\tau}_B} & 0 \\ 0 & 1 + e^{\mp i\boldsymbol{G}\cdot\boldsymbol{\tau}_B} \end{pmatrix},$$

where we introduce K-valleys momenta, $\boldsymbol{K}_\xi^{(j)} = \xi K[\cos(j2\pi/3), -\sin(j2\pi/3)]$, with $K = 4\pi/3a$ ($a \approx 2.46$ Å is the lattice constant of graphene). We note that for $\boldsymbol{r}_0 = (0, a/\sqrt{3})$ and $\boldsymbol{r}_0 = (0, -a/\sqrt{3})$, Eq. (2) transforms to the Hamiltonian of ABAC and ABCB, respectively. Likewise,

the Hamiltonian of monolayer-twisted-trilayer structures is given by

$$\mathcal{H}_{\mathrm{ABA+1}} = \begin{pmatrix} H_{\mathrm{g}}^{s} & V & \tilde{W} & 0 \\ V^{\dagger} & H_{\mathrm{g}}^{b} - \sigma_z \Delta' & V^{\dagger} & \mathcal{W}_{\mathrm{BA+1}}(\boldsymbol{r}_0) \\ \tilde{W}^{\dagger} & V & H_{\mathrm{g}}^{s} + \mathcal{E}_b & \mathcal{V}(\boldsymbol{r}_0) \\ 0 & \mathcal{W}_{\mathrm{BA+1}}^{\dagger} & \mathcal{V}^{\dagger}(\boldsymbol{r}_0) & H_{\mathrm{g}} + \mathcal{E}_t \end{pmatrix}, \tag{3a}$$

$$\mathcal{H}_{\mathrm{ABC+1}} = \begin{pmatrix} H_{\mathrm{g}}^{s} & V & W & 0 \\ V^{\dagger} & H_{\mathrm{g}}^{b} & V & \mathcal{W}_{\mathrm{AB+1}}(\boldsymbol{r}_0) \\ W^{\dagger} & V & H_{\mathrm{g}}^{s} - \sigma_z \Delta' + \mathcal{E}_b & \mathcal{V}(\boldsymbol{r}_0) \\ 0 & \mathcal{W}_{\mathrm{AB+1}}^{\dagger}(\boldsymbol{r}_0) & \mathcal{V}^{\dagger}(\boldsymbol{r}_0) & H_{\mathrm{g}} + \mathcal{E}_t \end{pmatrix}, \tag{3b}$$

$$\mathcal{W}_{\mathrm{BA+1}}(\boldsymbol{r}_0) = \frac{1}{6} \sum_{j=0}^{2} \begin{pmatrix} \gamma_2 e^{+i\xi \frac{2\pi}{3} j} & \gamma_2 e^{-i\xi \frac{2\pi}{3} j} \\ \gamma_5 & \gamma_5 e^{+i\xi \frac{2\pi}{3} j} \end{pmatrix} e^{-i\boldsymbol{K}_{\xi}^{(j)} \cdot \boldsymbol{r}_0}.$$

Convergence analysis

In our analysis, we compute the electron density in each layer by brute-force diagonalisation of the Hamiltonian (1) for a sufficiently dense grid of points in reciprocal space within a circular area with cut-off wavevector, k_c. For each nABAm film, as k_c increases, the ferroelectric polarisation converges to a P_z-value, displayed below in the left panel of Fig. 2 (c). To illustrate that this value of the cut-off is large enough, we consider behaviour of P_z versus k_c for two characteristic structures: 4ABA and 4ABA3, shown in Fig. 1.

The low-energy dispersion of 4ABA graphite film features a couple of flat bands and a pair of Dirac dispersive bands [see inset in Fig. 1 (a)]. The former, strongly localised at the bottom and twin boundary layers, induces a polarisation in the positive z-direction, while the latter, hybridising with deeper bands, contributed to P_z in negative direction of z-axis. As shown Fig. 1 (a), it is necessary to sample an area of $5 \cdot 10^{-2}$ Å^{-2} (or a value for the cut-off energy of ~ -0.13 eV) to reach convergence.

In contrast, the low-energy dispersion of 4ABA3 exhibits two pairs of flat bands [see inset in

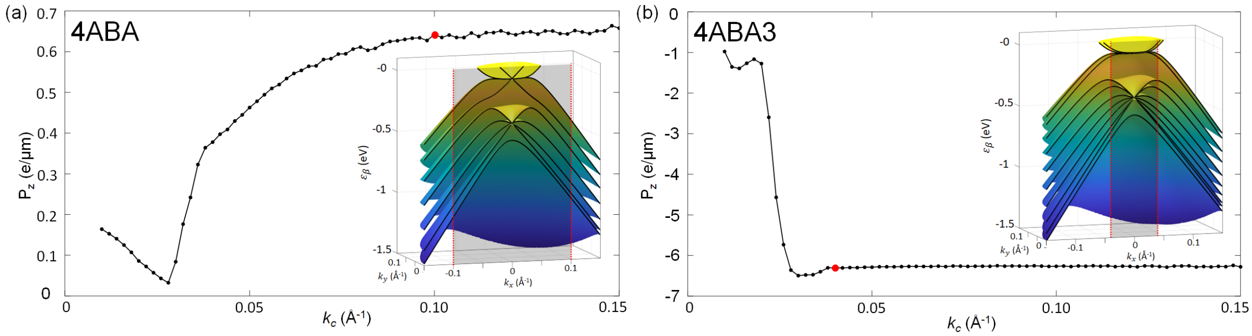

Figure 1: Value for the ferroelectric polarisation, P_z, as a function of the radius of the circle centred at $\boldsymbol{K}_{\pm}$ in reciprocal space, k_c taken to compute the electron density in each layer, for 4ABA (a) and 4ABA3 (b) tetralayer graphene. For the latter, it suffices to sample the area on which flat bands extend to obtained a converged value.

Fig. 1 (b)], with a much stronger localisation at the twin boundary and non-dimer site of surface layer, that are well-separated from the rest continuum of the deep-energy states. In this case P_z converges at smaller area of reciprocal space, about $5 \cdot 10^{-3}$ Å^{-2} (or a value for the cut-off energy of ~ -0.07 eV), which corresponds to the region of high density of states of the flat bands.

Self-consistent implementation of screening

Here, we extend the above-described SWMcC model to incorporate the effects of internal electric fields produced by the charge redistribution. These fields produce an energy offset in each layer, which can be captured in our model by adding a term $\epsilon_i \hat{1}_2$ to the i-th diagonal block of the Hamiltonians in Eqs. (1), (2) and (3) using the formula[6]

$$\epsilon_i - \epsilon_{i-1} = \frac{e^2 d}{2\varepsilon_0}\left[(n_i - n_{i-1})\frac{1+\varepsilon_z^{-1}}{2} + \sum_{j>i} n_j \varepsilon_z^{-1} - \sum_{j'<i-1} n'_j \varepsilon_z^{-1}\right], \quad (4)$$

with $d \approx 3.35$ Å being the interlayer distance in graphene stacks, $\varepsilon_z \approx 2.6$[6] the relative permittivity of graphene. We determine the Fermi level for neutral structures, and calculate the charge redistribution across the layers, taking into account only the eigenvectors associated to states below the Fermi level. Because the charge densities n_i also depend implicitly on the on-layer potentials,

the expression in Eq. (4) needs to be solved self-consistently. In the first iteration, we set all on-layer energies to zero, $\epsilon_i^{(0)} = 0$, which gives the value of polarisation for the unscreened system, P_z^u. In each iteration, we use Eq. (4) to compute a new set of on-layer energies, $\tilde{\epsilon}_i^{(n)}$, which are used to update the value employed in the following iteration using

$$\epsilon_i^n = \eta\tilde{\epsilon}_i^{(n)} + (1-\eta)\epsilon_i^{(n-1)},$$

where η determines the fraction of the value for the on-layer energies that is updated in each iteration, and was taken to be $4 \cdot 10^{-4}$ in our work. This linear-mixing algorithm is necessary in a broad range of self-consistent methods to avoid overshooting[7].

In Figs. 2(a) and 2(b), we show the value of P_z and electron densities in each layer as a function of the iteration for 1ABA and 4ABA3 films, highlighting importance of self-consistent calculations. Histograms in Fig. 2(c) show comparison of P_z values for mABAn films with less than 8 layers computed with (right) and without (left) account of self-consistent screening. We also note that our results strongly depend on the input parameters, and suggest that measuring P_z in these systems could be an experimental route to narrow down the possible values of the SWMcC parameters.

Analysis of parametric dependence

Equation (3) of the main text is based on the analysis shown in Fig. 3, where each panel represents dependence of P_z^u of ABCB tetralayer graphene on one of the SMWcC parameters. The blue dots are numerically computed values of P_z^u and the red lines are the linear interpolations, which we use to obtain the coefficients $\mathcal{X}_{4,D,2,5}$. We observe the linear dependence of P_z on the symmetry-breaking parameters v_4, Δ', γ_2 and γ_5. The middle and rightmost panels of Fig. 3 (a), shows the dependence on γ_1 and v, when v_4 is kept as the only non-zero electron-hole symmetry-breaking parameter, which is proportional to $\gamma_1|\gamma_1| \times v^{-3}$, respectively. Likewise, in the same panels of Figs. 3 (b), (c) and (d), we demonstrate that P_z^u as the power law $|\gamma_1| \times v^{-2}$ when Δ', γ_2 and γ_5

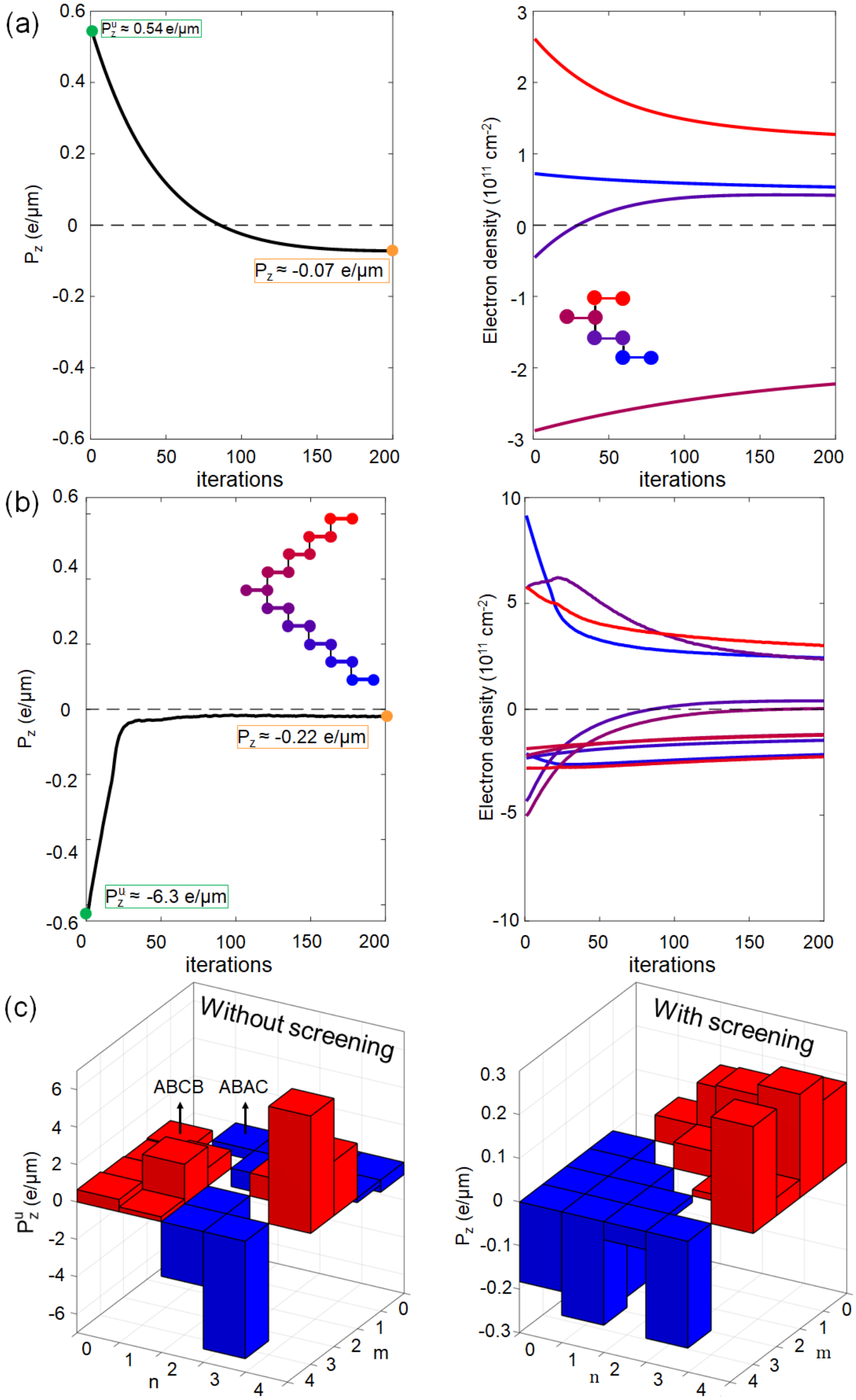

Figure 2: Convergence analysis of P_z and n_i for (a) 1ABA and (b) 4ABA3 films. The latter evidences that, for larger films, electron charge primarily redistributes to the surface and twin boundary layers. (c) Electric polarisation of mABAn, P_z^u (P_z), displayed in the form of a histogram, using a model that neglects (accounts for) screening effects of electron-density redistribution.

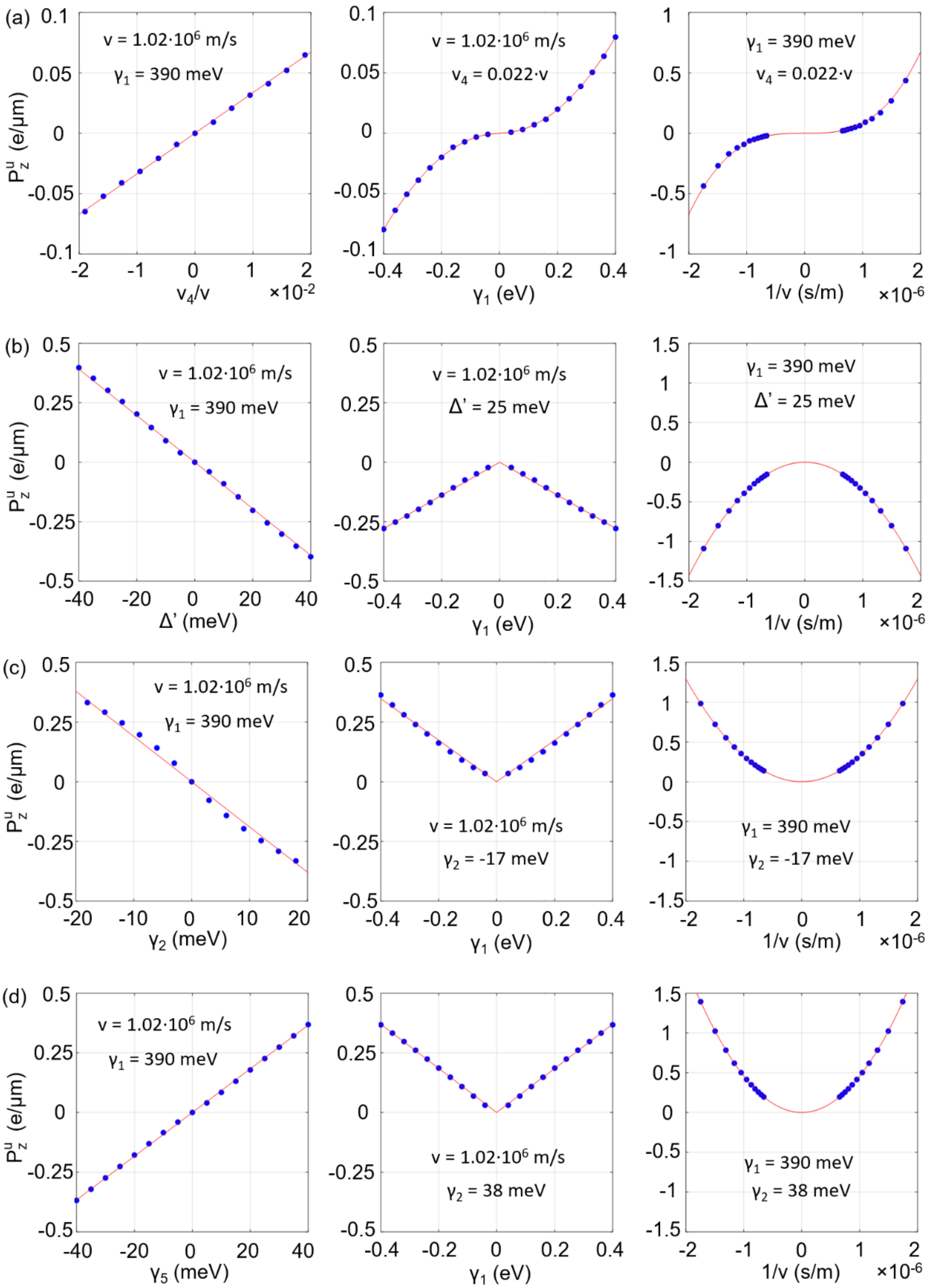

Figure 3: Numerical values (blue points) and fitting curves (red) computed to support the parametric dependence of the first, second, third and fourth terms in Eq. (2) in the main text are analysed in the triad of panels (a), (b), (c) and (d), respectively, for ABCB tetralayer graphene. In the leftmost panels, we demonstrate the linear dependence of each SWMcC parameter that induces ferroelec-

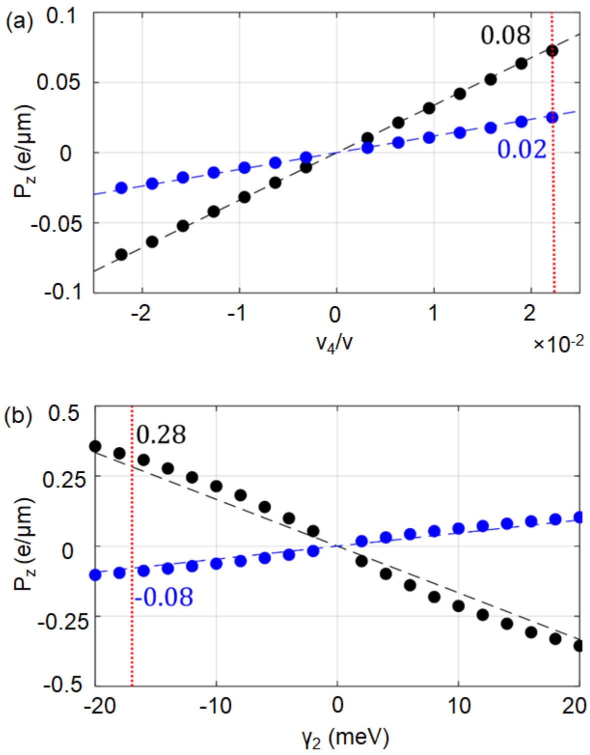

Figure 4: Dependence of P_z^u (black) and P_z (blue) as a function of each individual SWMcC parameter responsible for electron-hole asymmetry. Vertical red dotted lines lie on the values given in[4], which we use in our work. Adding all the values where they intersect the black and blue interpolating lines amounts approximately the value por P_z^u and P_z obtained using the full SWMcC model, and shown as the first and last values in Fig. 2(a).

are kept as the only non-zero parameters, respectively.

In Fig. 4, we extend analysis of P_z when effect of self-consistent screening is taken into account. In particular, we compare the leftmost panels of Fig. 3 using a model with (blue dots) and without (black dots) the effects of screening, and demonstrate that, while $\mathcal{X}_4$, $\mathcal{X}_D$ and $\mathcal{X}_5$ reduce their values by a factor of $\sim$4, 1.4 and 2.2, respectively, the coefficient $\mathcal{X}_2$ changes sign. As stated in the main text, this peculiar behaviour plays a prominent role in the change of sign of polarisation, being $P_z^u \approx 0.54\ \mathrm{e}/\mu\mathrm{m}$ in the unscreened model and $P_z \approx -0.07\ \mathrm{e}/\mu\mathrm{m}$ in the screened model [see Fig. 2(a)]. It is also worth mentioning that adding the individual contributions to P_z (or P_z^u) from the four symmetry-breaking terms amounts approximately to the to computed using the full SWMcC model with (without) the account for screening effects.

Lattice relaxation in twisted tetralayer structures

To describe lattice reconstruction of moiré superlattice formed at the twisted interface we introduce in-plane displacement fields $\boldsymbol{u}_{t/b}$ acting in top/bottom stacks. For 3+1 tetralayers top (bottom) stack consists of 1 (3) graphene layers, while for 2+2 structures the top/bottom stacks are graphene bilayers. To find $\boldsymbol{u}_{t/b}$ we minimize sum of elastic, $U = \sum_{l=t,b}\left[(\lambda_l/2)\left(u_{ii}^{(l)}\right)^2 + \mu_l u_{ij}^{(l)} u_{ji}^{(l)}\right]$, and local adhesion, $W\left(\boldsymbol{r}_0 = \theta\hat{z}\times\boldsymbol{r} + \boldsymbol{u}^{(t)} - \boldsymbol{u}^{(b)}\right)$, energies over supercell with periodicity of moiré superlattice given by twist angle θ expanding the displacement fields into Fourier series[8]: $\boldsymbol{u}_{t/b} = \sum_{\{\lambda\},j=0,1,2}\left[\hat{R}_{2\pi j/3}\boldsymbol{u}_{\{\lambda\}}^{(t/b)}\right]\sin\left(\boldsymbol{g}_{\{\lambda\}}^{(j,+)}\boldsymbol{r}\right)$ with $\boldsymbol{g}_{\{\lambda\}}^{(j,+)} = \theta\boldsymbol{G}_{\{\lambda\}}^{(j,+)}\times\hat{z}$ and $\hat{R}_\phi$ is counter clock-wise rotation on angle ϕ around $\hat{z}$. Obtained values of non-negligible Fourier amplitutes are listed in Table 1. In calculations, elastic moduli of top/bottom stacks were determined by those of graphene $\lambda_G = c_0 Y\nu/(1+\nu)(1-2\nu)$, $\mu_G = c_0 Y/2(1+\nu)$ (with Young module, $Y = 1\,$TPa, and Poisson ratio, $\nu = 0.19$[9]), multiplied by the number of graphene layers in each stack. For the adhesion energy at twisted interface we used the following expression[10,11]: $W(\boldsymbol{r}_0) = \sum_{\Lambda=1,2,3} w_\Lambda \sum_{j=0,1,2}\cos\left(\boldsymbol{G}_\Lambda^{(j,+)}, \boldsymbol{r}_0\right)$ with $w_1 = 0.775\,\mathrm{meV/A^2}$, $w_2 = -0.071\,\mathrm{meV/A^2}$, $w_3 = -0.018\,\mathrm{meV/A^2}$.

Table 1: Main non-zero Fourier series coefficients (in) of displacement fields describing reconstruction in twistronic tetralayers 3ABA+1, 3ABC+1 and 2AB+2BA structures with $\theta = 0.05°$, which were used in Fig. 2 of the main manuscript.

	3AB(A/C)+1		2AB+2BA
$\{\lambda_1, \lambda_2\}$	(1L) $u^{(t)}_{\lambda_1,\lambda_2}$	(3L) $u^{(b)}_{\lambda_1,\lambda_2}$	$u^{t}_{\lambda_1,\lambda_2}(= -u^{b}_{\lambda_1,\lambda_2})$
1, 0	(0.2898, 0.1687)	−(0.0979, 0.0565)	(0.1938, 0.1119)
2, 0	(0.1394, 0.0797)	−(0.0463, 0.0265)	(0.0910, 0.0526)
3, 0	(0.0875, 0.5089)	−(0.0292, 0.0169)	(0.0573, 0.0330)
4, 0	(0.0606, 0.0349)	−(0.0202, 0.0116)	(0.0389, 0.0225)
5, 0	(0.0428, 0.0247)	−(0.0143, 0.0082)	(0.0264, 0.0153)
6, 0	(0.0322, 0.0185)	−(0.0107, 0.0062)	(0.0201, 0.0116)
7, 0	(0.0227, 0.0132)	−(0.0076, 0.0044)	(0.0130, 0.0075)
8, 0	(0.0191, 0.0109)	−(0.0064, 0.0037)	(0.0121, 0.0070)

References

(1) McClure, J. W. Band Structure of Graphite and de Haas-van Alphen Effect. *Phys. Rev.* **1957**, *108*, 612–618.

(2) Slonczewski, J. C.; Weiss, P. R. Band Structure of Graphite. *Phys. Rev.* **1958**, *109*, 272–279.

(3) McClure, J. W. Theory of Diamagnetism of Graphite. *Phys. Rev.* **1960**, *119*, 606–613.

(4) Yin, J. et al. Dimensional reduction, quantum Hall effect and layer parity in graphite films. *Nature Physics* **2019**, *15*, 437–442.

(5) Garcia-Ruiz, A.; Deng, H.-Y.; Enaldiev, V. V.; Fal'ko, V. I. Full Slonczewski-Weiss-McClure parametrization of few-layer twistronic graphene. *Phys. Rev. B* **2021**, *104*, 085402.

(6) Slizovskiy, S.; Garcia-Ruiz, A.; Berdyugin, A. I.; Xin, N.; Taniguchi, T.; Watanabe, K.; Geim, A. K.; Drummond, N. D.; Fal'ko, V. I. Out-of-Plane Dielectric Susceptibility of Graphene in Twistronic and Bernal Bilayers. *Nano Letters* **2021**, *21*, 6678–6683, PMID: 34296602.

(7) Woods, N. D.; Payne, M. C.; Hasnip, P. J. Computing the self-consistent field in Kohn–Sham density functional theory. *Journal of Physics: Condensed Matter* **2019**, *31*, 453001.

(8) Enaldiev, V. V.; Zólyomi, V.; Yelgel, C.; Magorrian, S. J.; Fal'ko, V. I. Stacking Domains and Dislocation Networks in Marginally Twisted Bilayers of Transition Metal Dichalcogenides. *Phys. Rev. Lett.* **2020**, *124*, 206101.

(9) Androulidakis, C.; Zhang, K.; Robertson, M.; Tawfick, S. Tailoring the mechanical properties of 2D materials and heterostructures. *2D Materials* **2018**, *5*, 032005.

(10) Zhou, S.; Han, J.; Dai, S.; Sun, J.; Srolovitz, D. J. van der Waals bilayer energetics: Generalized stacking-fault energy of graphene, boron nitride, and graphene/boron nitride bilayers. *Phys. Rev. B* **2015**, *92*, 155438.

(11) Carr, S.; Massatt, D.; Torrisi, S. B.; Cazeaux, P.; Luskin, M.; Kaxiras, E. Relaxation and domain formation in incommensurate two-dimensional heterostructures. *Physical Review B* **2018**, *98*, 224102.
